# A Three-Dimensional Hyaluronic Acid-Based Scaffold Model for Electroporation-Enhanced Resveratrol Therapy in Triple-Negative Breast Cancer

**DOI:** 10.1007/s12013-026-02127-8

**Published:** 2026-08-14

**Authors:** Pragatheiswar Giri, Monica Dettin, Annj Zamuner, Maria Teresa Conconi, Ignacio G. Camarillo, Nicolò Martinelli, Luigi Dall’Olmo, Elisabetta Sieni, Raji Sundararajan

**Affiliations:** 1 School of Engineering Technology, Purdue University, West Lafayette, IN 47907 USA; 2 Department of Industrial Engineering, University of Padova, Padova, 35131 Italy; 3 Department of Civil, Environmental, and Architectural Engineering, University of Padova, Padova, 35131 Italy; 4 Department of Pharmaceutical and Pharmacological Sciences, University of Padova, Padova, 35131 Italy; 5 Dept. of Biological Sciences, Purdue University, West Lafayette, IN 47907 USA; 6 Department of Surgical, Oncological and Gastroenterological Sciences, University of Padova, Padova, 35128 Italy; 7 Dept. of Applied and Theoretical Science, University of Insubria, Varese, 21100 Italy

**Keywords:** 3D scaffold, Resveratrol, Electroporation, Triple-negative breast cancer, Reactive oxygen species

## Abstract

Triple-negative breast cancer (TNBC) is an aggressive malignancy defined by the absence of estrogen receptor (ER), progesterone receptor (PR), and human epidermal growth factor receptor 2 (HER2). These molecular characteristics render targeted therapies less effective and contribute to poor clinical outcomes. Towards this, we studied the electroporation-mediated delivery of resveratrol, a polyphenolic compound with pleiotropic anticancer activities, on MDA-MB-231, human TNBC cell line. Both conventional two dimensional (2D) monolayer cell culture and three-dimensional (3D) hyaluronic acid HA-EAbuK-IKVAV scaffold-based cell cultures were used. Two electroporation (EP) protocols (EP1: 8 pulses, 800 V/cm, 100 µs at 1 Hz; EP2: 8 pulses, 1000 V/cm, 100 µs at 1 Hz) were evaluated for their effects on cytotoxicity and reactive oxygen species (ROS) generation. In 3D, the combined treatment similarly reduced viability to 20% (EP1 + Resv) and 17% (EP2 + Resv), whereas in 2D, the viability reduction is 24% and 18%, respectively. Mechanistically, ROS levels increased substantially with the combination treatment: in 2D, ROS rose to 3 times (EP1 + Resv) and around 3.5 times (EP2 + Resv) the control, while in 3D, ROS increased to 2.4 times (EP1 + Resv) and 2.9 times (EP2 + Resv). Moreover, the combined treatment of electroporation and resveratrol administration on 3D HA-EAbuK-IKVAV-cultured cells decreased the expression of cell cycle regulatory protein Cdk2 and proliferation marker Ki67. These findings highlight the importance of 3D microenvironmental cues in modulating TNBC responsiveness to resveratrol and underscore the potential of combining electroporation with natural compounds as a rational therapeutic strategy for this challenging subtype of breast cancer.

**Supplementary Information:**

The online version contains supplementary material available at https://doi.org/10.1007/s12013-026-02127-8.

## Introduction

Triple-negative breast cancer (TNBC) accounts for approximately 15% of all breast cancer cases yet carries a disproportionate risk of relapse and metastasis due to its high proliferative index, genomic instability, and limited therapeutic targets [1–3]. Unlike ER-positive or HER2-positive malignancies, TNBC lacks established receptors for hormone- or anti-HER2–based interventions, frequently leading to aggressive disease progression and poor patient outcomes [4, 5]. Although systemic chemotherapy (e.g., taxanes, anthracyclines, and platinum agents) and emerging immunotherapies can yield initial responses, drug resistance and off-target toxicity continue to pose major clinical challenges [6–8]. Consequently, there is an urgent need for novel strategies that can selectively target TNBC cells while sparing healthy tissues. Hence, to address this need, this word investigates electroporation, combined with a natural compound Resveratrol, as a novel potential strategy.

Resveratrol (3,5,4′-trihydroxy-trans-stilbene), a stilbenoid naturally found in grapes, berries, and peanuts, has garnered attention in this context for its potent antioxidant, anti-inflammatory, and anticancer properties [9]. Mechanistically, resveratrol exerts pleiotropic actions on key oncogenic signaling pathways, including NF-κB, MAPK, PI3K/AKT, among others, regulating cellular proliferation, apoptosis, and stress responses [10, 11]. By enhancing intracellular reactive oxygen species (ROS) levels, resveratrol imposes oxidative stress in cancer cells, which are often vulnerable to redox imbalances [12, 13]. Furthermore, its favorable pharmacologic safety profile and capacity to interfere with multiple molecular targets have heightened interest in exploiting resveratrol as both a primary and adjuvant modality in oncology [14, 15]. This multifaceted mechanism of action may provide a unique therapeutic window in TNBC, where traditional mono-therapeutic approaches frequently fail to address the complex and adaptive nature of the disease.

Beyond small-molecule discovery, advanced cell culture models have gained traction in dissecting drug responses under conditions approximating the tumor microenvironment (TME). Conventional 2D monolayer cultures do not recapitulate the complex cell–extracellular matrix (ECM) architecture and hypoxic gradients of in vivo tumors [16–18]. Three-dimensional (3D) scaffolds, hydrogels, and organoids can more accurately replicate the TME, enhancing the predictivity of preclinical assays [19–21]. Hyaluronic acid (HA)-based scaffolds are particularly relevant to breast tumor biology, given the critical role of HA in modulating cell adhesion, proliferation, and migration [22, 23]. When combined with peptides such as IKVAV—derived from laminin—these scaffolds can foster tumor-like ECM conditions, thus providing improved translational relevance [24].

To enhance the Resv uptake, electroporation was used, as using high-intensity, short-duration electric pulses, electroporation transiently disrupts phospholipid bilayers, thereby facilitating enhanced intracellular drug delivery [25–27]. By drastically increasing membrane permeability, electroporation can raise local intracellular concentrations of therapeutic agents—sometimes by several orders of magnitude—while minimizing systemic exposure [28, 29]. This approach, commonly referred to as electrochemotherapy (ECT) when used with established anticancer drugs, has demonstrated promising clinical outcomes in melanoma, sarcoma, and other malignancies [30–32]. Notably, combining electroporation with natural or repurposed agents (e.g., resveratrol) is gaining research momentum, as it may harness synergistic cytotoxic effects without the toxicity profile of more conventional chemotherapeutics [33, 34].

In this study, we detail a 3D HA-EAbuK-IKVAV scaffold model to evaluate the therapeutic potential of resveratrol, combined with electroporation, in TNBC cells. MDA-MB-231 cells were cultured in 3D scaffolds and compared to their 2D counterparts. Two electroporation protocols featuring different electric-field strengths and pulse durations were tested to delineate their distinct effects on membrane permeabilization and cell killing. Post-treatment assessments focused on viability and intracellular ROS levels. Our results suggest that a 3D microenvironment significantly enhances resveratrol’s electroporation-mediated cytotoxicity in TNBC, implicating this approach as a viable avenue for improved localized treatments and drug-delivery paradigms.

## Materials and Methods

### Cell Line and Culture

MDA-MB-231 (ATCC^®^ HTB-26™) cells, a well-established human TNBC line, were maintained in Dulbecco’s Modified Eagle Medium (DMEM, Thermo Fisher Scientific) supplemented with 10% fetal bovine serum (FBS) and 1% penicillin–streptomycin at 37 °C, 85% humidity, and 5% CO₂ [35]. Cells were passaged upon reaching 90% confluence. All experiments consider cells in their exponential-growth phase.

### Resveratrol Preparation

Resveratrol (≥ 99% purity; Sigma-Aldrich) was prepared as a concentrated stock in dimethyl sulfoxide (DMSO) to a final working concentration of 100 µM [36]. The final vehicle concentration was maintained at ≤ 0.1% (v/v) DMSO in all conditions, with a matched DMSO-only control included. Fresh working solutions were prepared immediately before each experiment to minimize degradation.

### 3D Scaffold Fabrication

EAbuK-IKVAV peptide was synthesized as described by established protocols [24]. In brief, EAbuK-IKVAV (7.2 mg) was dissolved in Milli-Q water (4 mL) and HA (molecular weight: 1000–125 kDa, Contipro a.s., 144 mg) was solubilized separately in Milli-Q water (8mL) under magnetic stirring and sonication. The peptide solution was added dropwise to the HA solution under sonication. The mixture was dispensed into 48-well plates (~ 320 mg per well), frozen in liquid nitrogen, and lyophilized to create porous scaffolds. Crosslinking occurred with 60 mM N-(3-dimethylaminopropyl)-N′-ethylcarbodiimide (EDC) in an EtOH: Milli-Q (95:5 v/v) solution for 24 h at 4 °C [24]. Finally, the scaffolds were rinsed extensively and lyophilized again.

### Electroporation Protocols

Electroporation was conducted using a BTX ECM-830 square-wave electroporator (Genetronics Inc.) [25]. Two distinct pulse configurations in the field of reversible electroporation following ESOPE suggestions [34, 36] were applied:

1. **EP1**: Eight unipolar square-wave pulses at 800 V/cm for 100µs each, at 1 Hz.
2. **EP2**: Eight unipolar square-wave pulses at 1000 V/cm for 100µs each, at 1 Hz.

A parallel-plate electrode interfaced with an 8-well slide chamber (1 cm × 1 cm per well). This setup facilitated consistent electric field distribution and minimized cross-contamination.

### Electroporation Protocols for permeabilization

Cell membrane permeabilization was tested in previous experiments using cells cultured in HA-EAbuK-IKVAV or in 2D adhesion condition, and without resveratrol. The cells were using the propidium iodide, PI (Sigma Aldrich, Saint Louis, MO, USA), and Hoechst 33,342, HOE (ThermoFisher, Waltham, MA, USA), two fluorescent dyes. PI penetrates cell membrane of death or damaged cells or permeabilized cell [37, 38], and it is an indicator of membrane permeabilization. Whereas HOE stain all cells in culture. The fluorescence was observed using Olympus IX51 inverted microscope setting PI excitation at 538 nm with emission around 619 nm and HOE excitation at 352 nm, with emission around 455 nm. Electroporation was conducted using the EPS-02 square-wave pulse generator (Igea S.p.A, Carpi (MO), Italy) using same electric field-electrode and well conditions, i.e. 8 unipolar square-wave pulses at 800 V/cm or 1000 V/cm, as in electroporation with resveratrol experiments.

### 3D vs. 2D Culture Setup

**Fig. 1:**
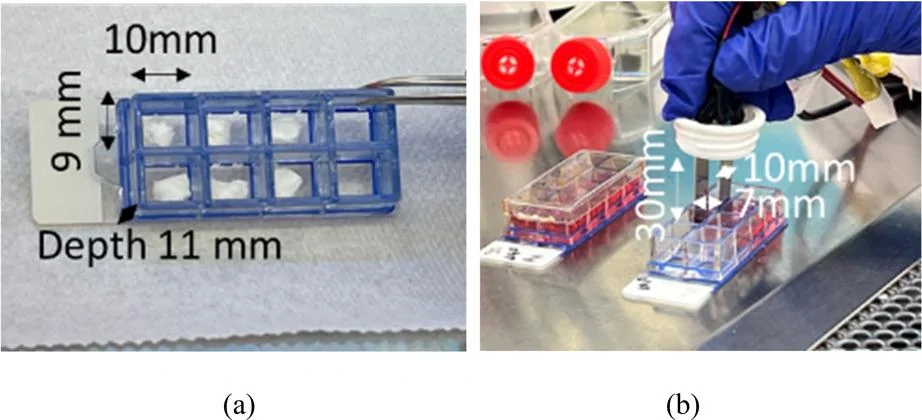
Experimental equipment and setup. (**a**) Matrices of HA-IKVAV 3D scaffolds (dry) after lyophilization. (**b**) Electroporation using the BTX ECM-830 on the incubated 3D cell cultures sample, 8-well rectangular chamber (each 1 cm x 1 cm surface area), Parallel plate electrode (distance between the plates is 7 mm)

For 3D cultures, each lyophilized HA-EAbuK-IKVAV scaffold was hydrated with 180 µL of cell culture medium and incubated for 1 h in a chamber slide with 8 wells (sized 9 × 10 × 11 mm). Subsequently, 20 µL of MDA-MB-231 cell suspension (9 × 10⁶ cells/mL) was seeded onto the scaffold, followed by the addition of 300 µL of fresh medium after 1 h, yielding a total volume of 500 µL per well [39]. After 24 h the scaffold became transparent. After 72 h to permit scaffold infiltration and cell–ECM interactions, electroporation (EP1 or EP2) was performed in the presence or absence of 100 µM resveratrol. After treatment the cultures were incubated 24 h before the viability assay. Figure 1 displays the experimental setup.

For 2D cultures, cells were seeded at equivalent densities (final volume 500 µL per well) on a standard tissue-culture surface and subjected to identical electroporation parameters and treatment regimens. Both 2D and 3D samples were incubated for 24 h post-electroporation before further analysis.

### Cell Viability Assay

Cell viability was quantified using the RealTime-Glo™ MT assay (Promega), which measures ATP-driven luminescence as a proxy for metabolically active cells [40]. Post-treatment, 20 µL of electroporated cell suspension (1 × 10⁶ cells/mL) was seeded into 96-well plates with 55 µL culture medium. A 25 µL mixture of RealTime-Glo™ reagents was added per well, and luminescence was recorded at 24 h using a GloMax^®^ microplate reader (Promega). Results were normalized to untreated controls set at 100%.

### ROS Quantification

Intracellular ROS levels were evaluated using the ROS-Glo™ H₂O₂ assay (Promega) [41]. Following 18 h of incubation post-treatment, 20 µL of the supplied H₂O₂ substrate solution was added to each well. After 6 h at room temperature, 100 µL of detection reagent was introduced. Luminescence readings were acquired after 20 min using a GloMax^®^ reader. Elevated luminescent signals signify higher intracellular H₂O₂ and, by extension, elevated ROS generation. Data were normalized to control samples.

### Histology and Scaffold Morphology

The scaffold structure without cells was visualized through an Environmental Scanning Electron Microscope (ESEM) Quanta 200 (FEI, Hillsboro, OR, USA).

Scaffolds containing cells were fixed in 4% formaldehyde for 1 h and air-dried. Masson’s trichrome staining (Bio-Optica, Milan, Italy) was performed to visualize collagen-like matrix deposition and cell distribution [24, 42, 43]. Stained samples were imaged using an optical microscope (Zeiss Axiolab, Carl Zeiss AG, Oberkochen, Germania).

### Polymerase Chain Reaction (PCR)

Twenty-four hours after treatment, cells were pelleted and RNA extracted using the Monarch Spin RNA Isolation Kit (New England Biolabs) following manufacturer instructions. The RNA was quantified with Nanodrop Spectrophotometer (NanoDrop One, Thermo Fisher Scientific, USA). RNA was retro-transcripted to cDNA using High-Capacity RNA-to-cDNA kit (Applied Biosystems, Thermo Fisher Scientific, US) following manufacturer instructions. Qualitative PCR was performed as follow: a mix was prepared with 10 µL of 2X Quick Taq HS DyeMix Master Mix (Toyobo Co. Ltd., Osaka, Japan), 0.4 µL for each forward and reverse primers (obtained from S.I.A.L. srl, Rome, Italy) and appropriate volume for 25 ng of cDNA; nuclease-free water was added to a final volume of 20 µL for each reaction. The expression of col1a1 (F 5’-CCAGCCACCTCAAGAGAAGG-3’, and R 5’- CCGAACCAGACATGCCTCTT-3’) and col4a1 (F 5’-ATGTGACTGCCATGGAGTGA-3’ and R 5’- ACCCTTCATCCCTGGTAAGC-3’) genes for collagen type I and IV, respectively, was evaluated (housekeeping gene GAPDH, F 5’-CCATCTTCCAGGAGCGAGAT-3’, and R, 5’-GGTCATGAGTCCTTCCACGA-3’). The PCR program consists of initial pre-denaturation phase at 94 °C for 2’ followed by 30 cycles of 30’’ at 94 °C (denaturation), 30’’ at 56 °C (annealing), 1’ at 68 °C (extension). The final step was 5’ at 68 °C followed by 4 °C maintenance. The PCR was carried out using the Veriti 96-well thermal cycler (Applied Biosystems, Thermo Fisher Scientific, US). The PCR product was loaded in 2% agarose gel stained with SYBR Safe (Thermo Fisher Scientific, US) and electrophoresis was performed at 120 V for 40’. Gel was acquired with ChemiDoc Imaging System (Bio-Rad Laboratories, Inc., USA).

### Real-Time Quantitative Polymerase Chain Reaction (qPCR)

Total RNA was extracted from cultured cancer cells using the Monarch Spin RNA Isolation Kit (New England Biolabs) following manufacturer instructions. RNA was quantified with Nanodrop Spectrophotometer and converted to cDNA using High-Capacity RNA-to-cDNA kit (Applied Biosystems, Thermo Fisher Scientific, US), a concentration of 1ng/µL of cDNA was used for qPCR. Quantitative PCR was performed using TaqMan Fast Advanced Master Mix (Applied Biosystems, Thermo Fisher Scientific, US) in a QuantStudio 5 System (Thermo Fisher Scientific, US). TaqMan probes has been used: ACTB (Hs01060665_g1), TP53 (Hs01034249_m1), BCL-2 (Hs04986394_s1), MKI67 (Hs04260396_g1), CDK2 (Hs01548894_m1), COL4A1 (Hs00266237_m1). Quantitative PCR was performed using the following program: 50 °C for 2 min, 95 °C for 20 s, 40 cycles at 95 °C for 1s and 40 cycles at 60 °C for 20 s, followed by a melting curve. Relative RNA expression was evaluated after normalization for ACTB using the ∆∆CT method. Each experiment was performed in triplicate.

### Statistical Analysis

All experiments were conducted in triplicate, and each data point represents the mean ± standard error (SE). One-way ANOVA with Tukey’s multiple comparison post hoc analysis was used to identify significant differences among groups. Statistical calculations were performed with GraphPad Prism (v10.1.1), with *p* < 0.05 deemed statistically significant [44, 45].

## Results

### Scaffold Integrity and Cellular Organization

**Fig. 2:**
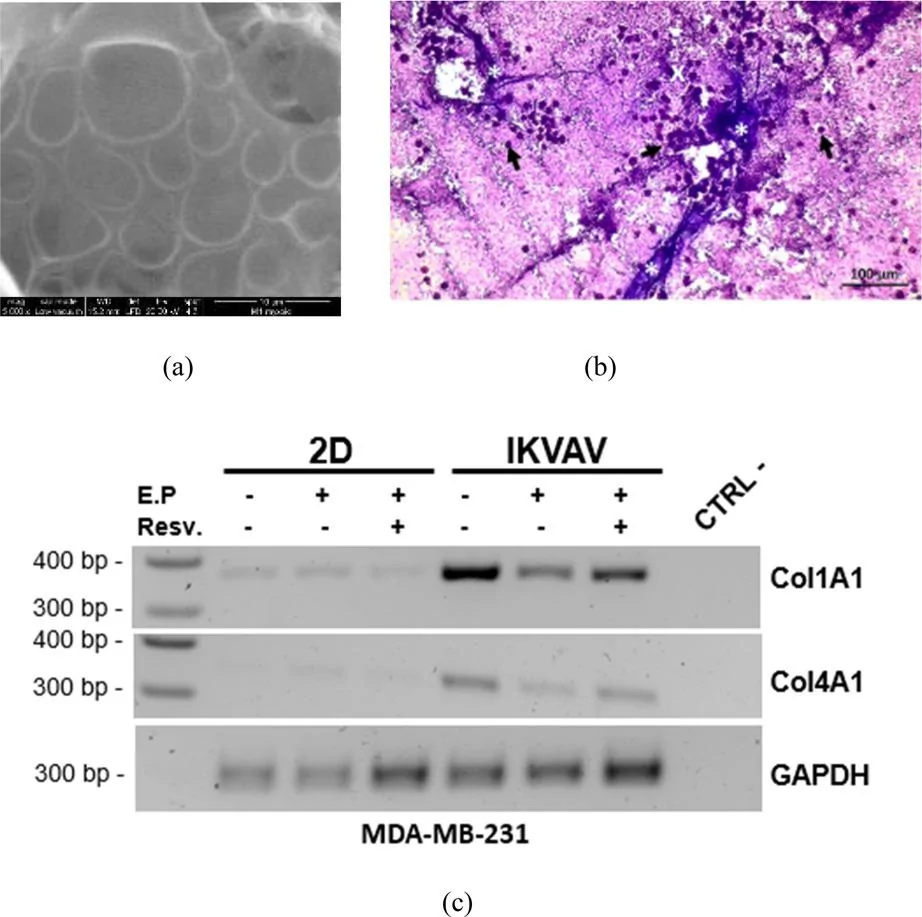
(**a**) ESEM image of the HA-EAbuK-IKVAV scaffold (Scale bars: 10 μm). (**b**) Histology staining, Masson trichrome: blue area, near white stars and white cross, are collagen area of the MDA-MB-231 cultured in the scaffold at 1000 μm, black arrow are cells. (Scale bars: 100 μm). (**c**) gel electrophoresis of PCR products shows col1a1 and col4a1 collagen expression in 2D and 3D culture (HA-EAbuK-IKVAV), GAPDH as housekeeping gene

ESEM micrographs (Fig. 2a) shows the characteristic porous morphology of the HA-EAbuK-IKVAV scaffolds, consistent with prior reports on functionalized with adhesion sequence polysaccharide–peptide matrices [22, 24]. Masson’s trichrome staining revealed that MDA-MB-231 cells were embedded within a collagen-like matrix, reflecting the capacity of the scaffold to replicate key aspects of the in vivo tumor microenvironment [46]. The organization of cells within the pores suggested robust cell–ECM interactions, a hallmark of 3D culture models.

### Culture Morphology

Masson’s trichrome staining was performed to visualize collagen-like matrix deposition and cell distribution [43, 47, 48], as shown in Fig. 2b: black arrows marks cell (violet), white stars highlight collagen fiber stained in blue and white cross the not-fibrous collagen stroma (in blue).

The collagen in the 3D cell culture was confirmed by PCR results related to 2D and 3D culture in Fig. 2c (fragment of examined sequence are between 300 and 400 bp long). From PCR data, MDA-MB-231 cells cultured in HA-EAbuK-IKVAV scaffolds had a higher expression of col1a1 and col4a1 genes compared to standard 2D culture. In particular, col1a1 is more expressed in the cells cultured in scaffolds.

### Electroporation of Cell Cultures

**Fig. 3:**
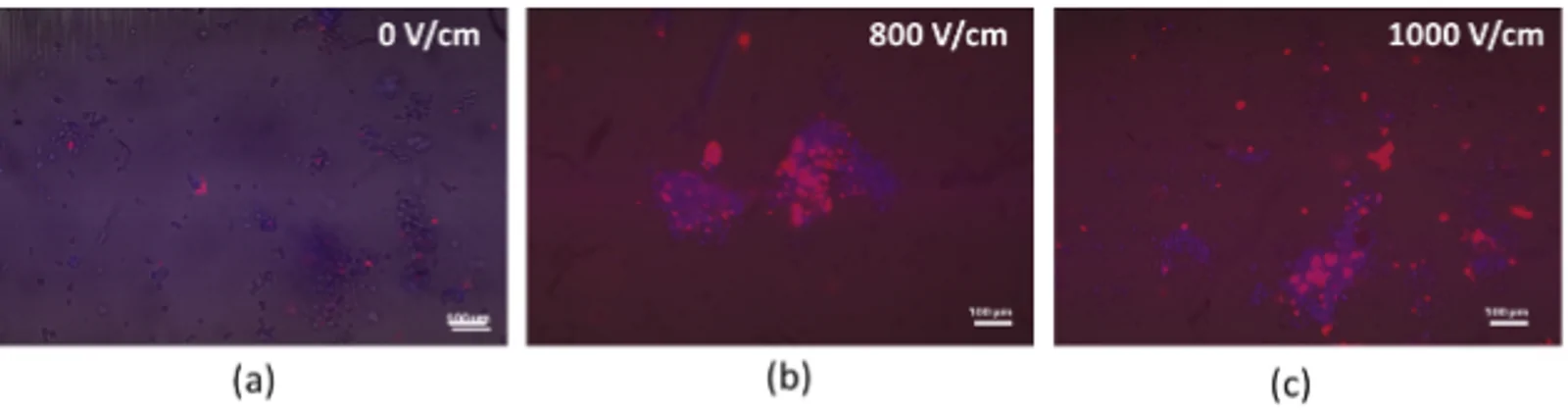
Representative fluorescence images of HA-EAbuK-IKVAV culture MDA-MB 231 at (**a**) 0 V/cm, (**b**) 800 V/cm and (**c**) 1000 V/cm (Scale bars: 100 μm). Red is due to Propidium Iodide fluorescence that marks electroporated cells. Whereas blue is due to Hoestch 33,342 that marks all cells

**Fig. 4:**
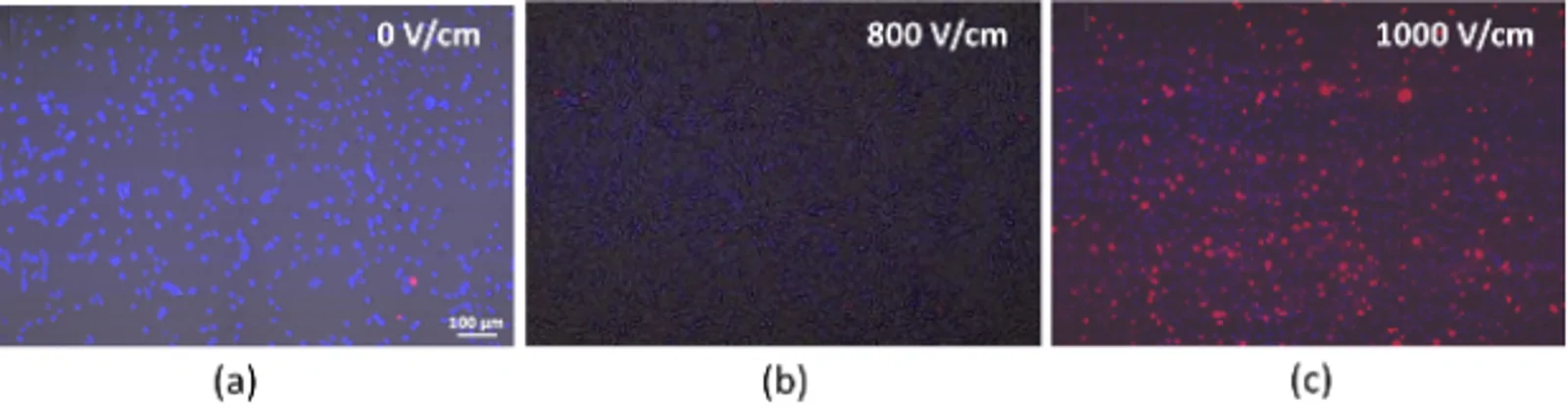
Representative fluorescence images of 2D culture MDA-MB 231 at (**a**) 0 V/cm, (**b**) 800 V/cm and (**c**) 1000 V/cm (Scale bars: 100 μm). Red is due to Propidium Iodide fluorescence that marks electroporated cells. Whereas blue is due to Hoestch 33,342 that marks all cells

Electroporation experiments show how much the 2 applied electric field, i.e., 800 and 1000 V/cm, are able to permeabilize cell membrane. Figure 3 displays a representative image of the 3D HA-EAbuK-IKVAV scaffolds cell cultures, observed after 0 V/cm, 800 V/cm, and 1000 V/cm electroporation treatments, in the absence of resveratrol, showing the sole cell membrane permeabilization efficiency. Figure 4 shows the representative image of the cells subjected to the same experiments for cell cultured just in adhesion electroporated in presence of cell medium at the same electric field strength than 3D cultures. In cells cultured in 3D scaffolds (Fig. 3) the electropermeabilization marked by PI, red, starts at a lower electric field, 800 V/cm, with respect to cells cultured in 2D conditions, for which partial electroporation, around 40–50% of red cells, appears at 1000 V/cm.

### Viability in 3D vs. 2D Under Electroporation and Resveratrol

**Fig. 5:**
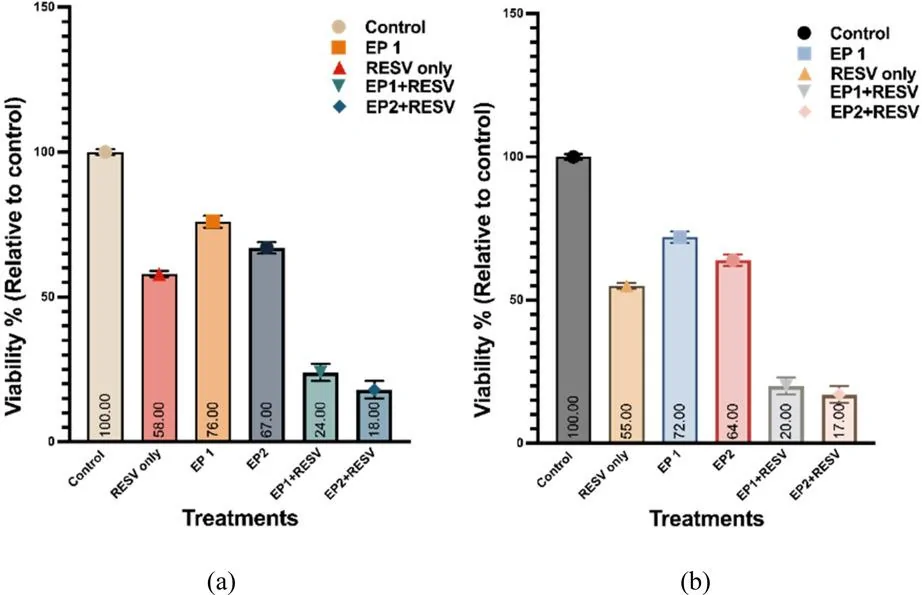
Cell viability results for EP1, EP2, Resv, EP1 + Resv, and EP2 + Resv conditions in both 2D cell culture (Fig. 5a) and 3D scaffold (Fig. 5b) with EP parameters EP1: 8 pulses, 800 V/cm, 100 µs at 1 Hz; EP2: 8 pulses, 1000 V/cm, 100 µs at 1 Hz. The error bars represent the standard error (SE)

Viability data at 24 h post-treatment shown in Fig. 5 underscored the potentiating effect of electroporation on resveratrol cytotoxicity, with marked differences observed between the 3D scaffold and 2D monolayers with lower viability in 3D than in 2D. With EP1 or EP2 alone, cell viability ranged from 67 to 76% of control, indicating moderate impact from membrane permeabilization, while Resveratrol alone (100 µM) indicated 55–58% survival, consistent with the known cytostatic properties of resveratrol. However, there is drastic reduction with EP1 + Resv, in both 3D and 2D. The viability dropped to 20% in 3D and to 24% in 2D for EP1 + Resv, while it was further lower, with 17% in 3D and to 18% in 2D.

In both cases, the difference between 2D and 3D is minimum and in the variability bar indicates that the two experiments could be considered comparable. Electroporation in the absence of resveratrol also showed a higher effect in 3D, suggesting that scaffolds increases the electric field effect and also it amplifies the electroporation efficacy (as shown in Figs. 3 and 4), possibly through altered cell polarity, membrane curvature, or local field distribution [25, 49]. Statistical analysis confirmed that all treatments significantly reduced cell viability compared with untreated control in both 2D and 3D cultures. In 2D cultures, the p values versus control were: EP1, *p* = 1.41 × 10⁻⁷; EP2, *p* = 3.67 × 10⁻⁹; RESV only, *p* = 2.19 × 10⁻¹⁰; EP1 + RESV, *p* = 1.93 × 10⁻¹³; and EP2 + RESV, *p* = 7.69 × 10⁻¹⁴. In 3D cultures, the p values versus control were: EP1, *p* = 2.44 × 10⁻⁸; EP2, *p* = 1.33 × 10⁻⁹; RESV only, *p* = 9.73 × 10⁻¹¹; EP1 + RESV, *p* = 1.04 × 10⁻¹³; and EP2 + RESV, *p* = 6.64 × 10⁻¹⁴. These results confirm that electroporation significantly potentiated resveratrol-mediated cytotoxicity in both culture models.

### Resveratrol-Induced ROS Accumulation

**Fig. 6:**
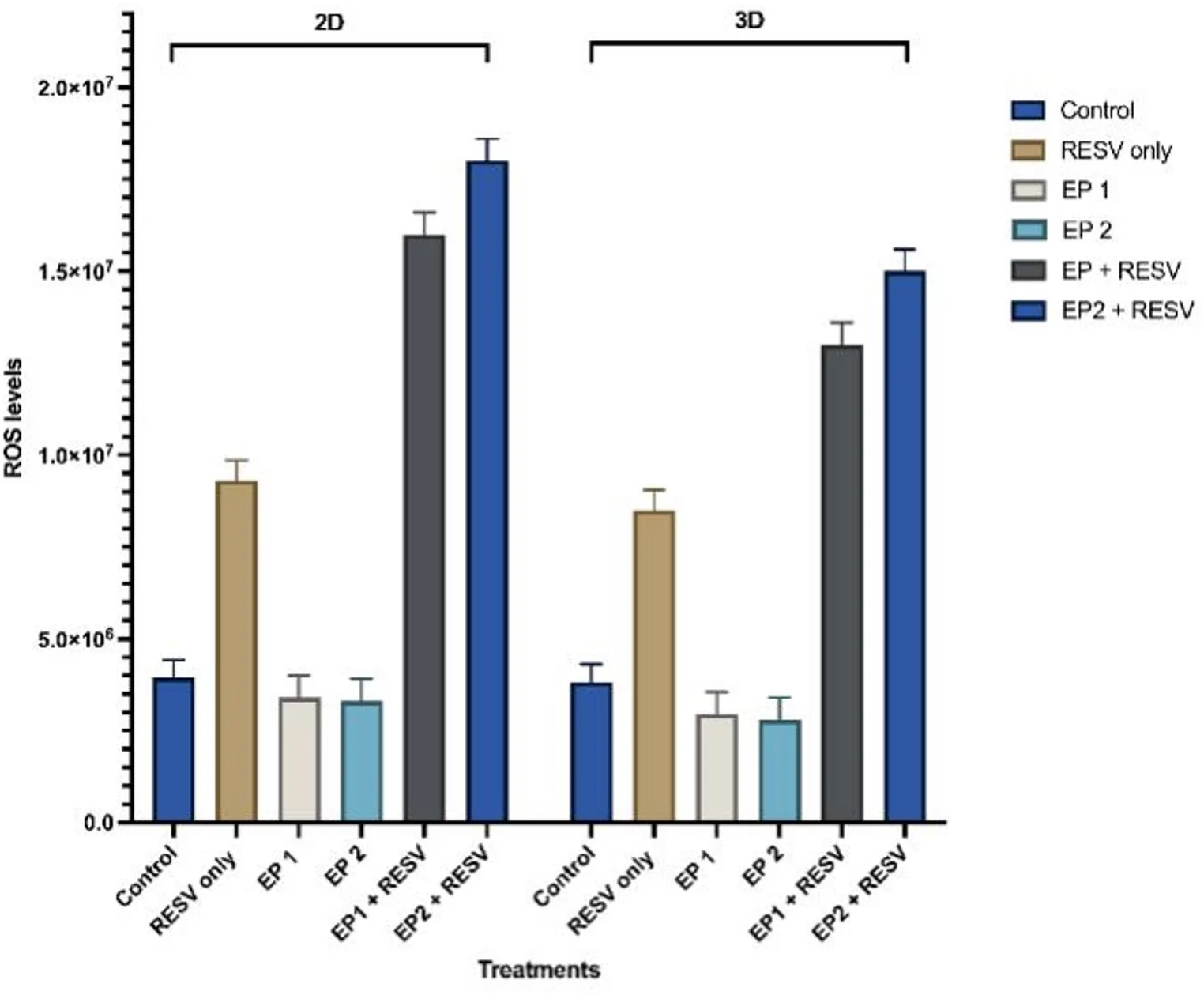
Reactive oxygen species (ROS) assay results for EP1, EP2, Resv, EP1 + Resv, and EP2 + Resv conditions in both 2D and 3D scaffold cell culture. (EP1: 8 pulses, 800 V/cm, 100 µs at 1 Hz; EP2: 8 pulses, 1000 V/cm, 100 µs at 1 Hz). The error bars represent the standard error (SE)

ROS levels, measured by H₂O₂-dependent luminescence, were significantly higher in groups treated with both electroporation and resveratrol. In 2D cultures, EP1 + Resv elevated ROS to ~ 305% relative to control, whereas EP2 + Resv was closer to ~ 356%. Resveratrol alone induced a moderate ROS increase (~ 135%). Combinatorial treatments (EP1 or EP2 plus resveratrol) drove ROS the maximum above control in 2D shown in Fig. 6.

In 3D scaffolds, baseline ROS production (untreated) was similar to 2D controls, but after treatment, the synergy in EP1 plus resveratrol pushed ROS levels to ~ 239%, the show an effect related to Rev combined to electroporation, nevertheless this effect seems lower among all groups. EP2 plus resveratrol was higher (~ 292%), yet still not above all individual treatments. These less elevated ROS measurements correlate with difference in culture conditions. Even if with less ROS level cancer cells experience irreversible oxidative stress when challenged by resveratrol’s redox-modulating properties in tandem with electroporation-facilitated uptake [12, 50]. Statistical analysis of ROS levels showed significant differences among treatment groups. In 2D cultures, the p values versus untreated control were: RESV only, *p* = 9.88 × 10⁻⁷; EP1, *p* = 0.840; EP2, *p* = 0.733; EP1 + RESV, *p* = 8.78 × 10⁻¹¹; and EP2 + RESV, *p* = 1.43 × 10⁻¹¹. In 3D cultures, the p values versus untreated control were: RESV only, *p* = 4.33 × 10⁻⁶; EP1, *p* = 0.457; EP2, *p* = 0.305; EP1 + RESV, *p* = 2.16 × 10⁻⁹; and EP2 + RESV, *p* = 2.14 × 10⁻¹⁰. These results indicate that resveratrol significantly increased ROS levels, and that the combination of electroporation with resveratrol produced the strongest ROS elevation in both 2D and 3D cultures.

### Real-Time PCR

**Fig. 7:**
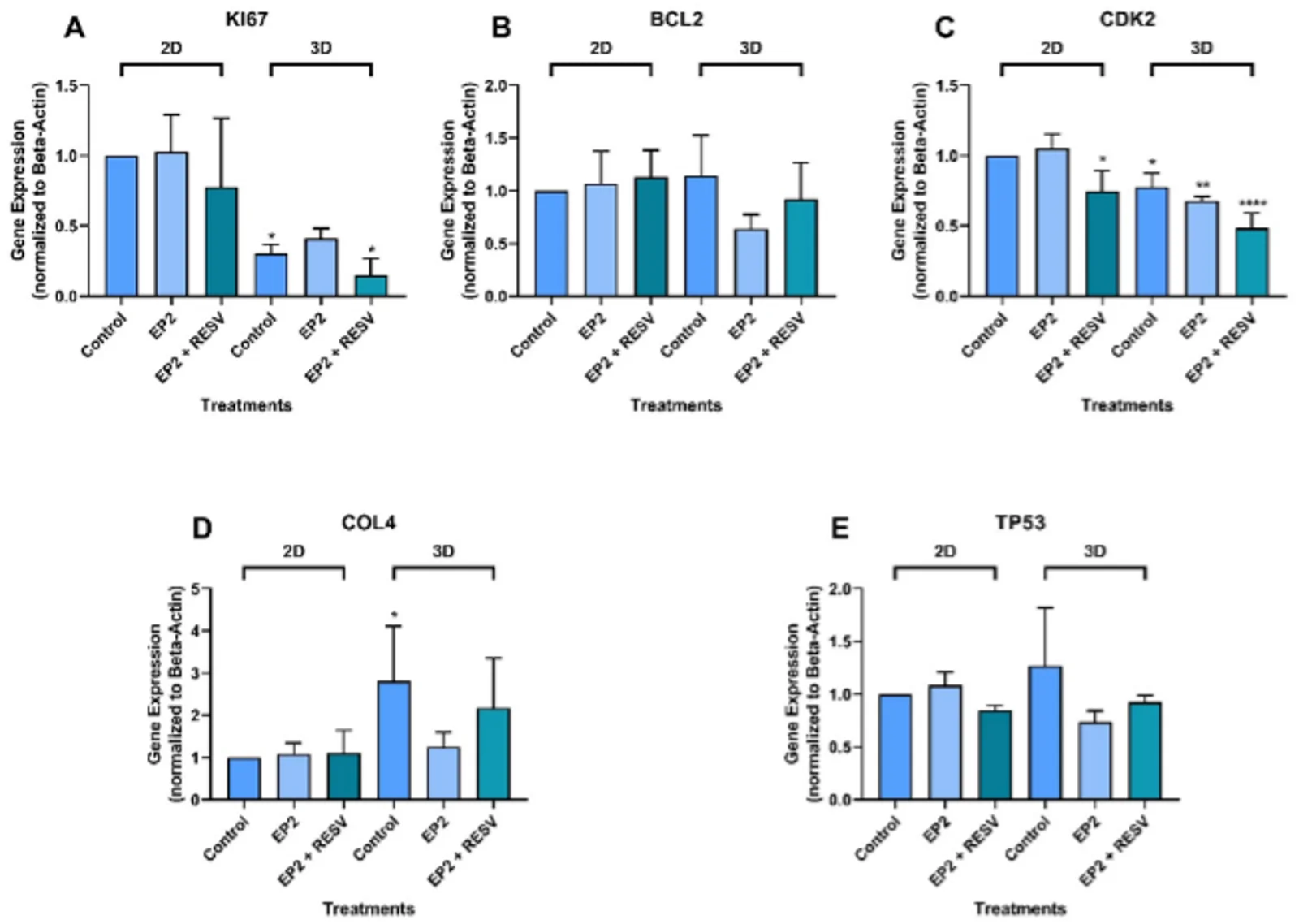
RT-qPCR analysis of gene expression in MDA-MB-231 cells after electroporation ± resveratrol in 2D and 3D HA-EAbuK-IKVAV scaffolds. Relative mRNA levels of (**A**) KI67, (**B**) BCL2, (**C**) CDK2, (**D**) COL4A1, and (**E**) TP53 are shown for untreated and treated groups (2D vs. HA-EAbuK-IKVAV-3D) with EP (1000 V/cm) (EP2) alone or EP + resveratrol (100 µM) (EP2 + RESV). Expression was normalized to β-actin and plotted relative to untreated 2D control. The data are presented as means ± SD. ∗*p* < 0.05, ∗∗*p* < 0.01 and ∗∗∗∗*p* < 0.0001. Statistical significance was determined by ordinary one-way ANOVA

Quantitative real-time PCR analysis was performed to assess the transcriptional regulation of key molecular markers in MDA-MB-231 triple-negative breast cancer (TNBC) cells following treatment (Fig. 7). The PCR profile indicates that electroporation (EP) engages a coupled stress-adaptation and microenvironmental transcriptional program in MDA-MB-231 cells that is strongly conditioned by 3D HA-EAbuK-IKVAV scaffold. In 2D cell culture the expression of MKI67 is similar for electroporated and not electroporated cells, whereas in the 3D IKVAV scaffold this proliferative signature is comparatively constrained, reflecting architecture- and adhesion-dependent control of cell-cycle (Fig. 7A). Moreover, EP within 3D scaffold leads to a reduction in the expression of BCL2 while the treatment with electroporation and resveratrol significantly reduces the expression of CDK2 both in 2D and 3D cell cultures compared to untreated 2D cultured cells, which is coherent with a reduced cell proliferation rate (Fig. 7B-C).

The increased expression of COL4A1 in untreated 3D HA-EAbuK-IKVAV cultured cells compared to untreated 2D cells further supports activation of an ECM remodeling/reciprocity axis, reflecting adaptation to scaffold residence and/or EP-associated mechanical and oxidative cues that can modulate basement-membrane gene programs and matrix deposition pathways (Fig. 7D). TP53 transcripts change only modestly across conditions, which is mechanistically coherent because p53 output is dominantly governed by protein stabilization, phosphorylation/acetylation dynamics, and MDM2-mediated turnover, rendering mRNA abundance an incomplete surrogate for pathway activation (Fig. 7E); hence, definitive attribution of p53-axis engagement requires orthogonal protein-level validation (p53, p21/CDKN1A, γH2AX) and apoptotic execution readouts (cleaved PARP, caspase-3/7).

Notably, inclusion of resveratrol with EP tends to attenuate EP-only pro-growth/pro-survival deviations toward baseline across several targets, aligning with a model in which EP enhances intracellular delivery of resveratrol and biases the response away from compensatory recovery and toward stress-intolerant cytotoxic trajectories. On the contrary, the 3D scaffold reveals these balances under tissue-relevant adhesion and diffusion constraints that are systematically underestimated in monolayer culture. Importantly, the tendency for EP + resveratrol to counterbalance EP-only deviations toward baseline across multiple targets suggests that enhanced intracellular resveratrol delivery shifts the system from an adaptive repair trajectory toward a more stress-intolerant state, through redox–metabolic perturbation and attenuation of compensatory survival signaling an effect that is most credibly interpreted in 3D where diffusion barriers, ligand density, and cell–ECM reciprocity collectively define therapeutically relevant response thresholds.

## Discussion

### 3D Cultures in TNBC Research

Triple-negative breast cancer (TNBC) is particularly notorious for its aggressive behavior and propensity for therapeutic resistance, phenomena that are closely linked to its unique TME [16, 51]. Indeed, microenvironmental factors such as ECM composition, cellular heterogeneity, and dynamic oxygen gradients shape tumor progression and therapeutic outcomes. Traditional 2D cultures, while foundational for early drug-screening workflows, fail to accurately recapitulate these spatial and compositional complexities. Consequently, 2D models often misrepresent drug efficacy and metabolic responses, leading to translational gaps when compounds move into in vivo or clinical testing.

In the present study, the hyaluronic acid HA-EAbuK-IKVAV scaffold was engineered to mimic the collagen-dense stroma frequently observed in breast tumors, specifically TNBC [22]. The IKVAV peptide sequence, derived from laminin, provides integrin-binding sites, thereby enhancing the physiologically relevant cell–ECM and integrin-mediated signaling axes. This design is critical, as integrin clustering and downstream focal adhesion kinase (FAK) activation have well-documented roles in modulating cellular proliferation, motility, and therapy resistance [18–20]. By adopting this 3D matrix, we more faithfully recapitulate biomechanical cues and nutrient diffusion gradients, permitting tumor cells to exhibit morphological and phenotypic traits akin to in vivo growth patterns.

Moreover, the 3D scaffold fosters pathological-relevant gradients of oxygen and nutrients, which can differentially activate hypoxia-inducible factor-1α (HIF-1α) and other stress-responsive pathways. These pathways, in turn, coordinate metabolic rewiring (e.g., partial or complete reliance on glycolysis) and adaptive survival mechanisms. As such, drug sensitivity profiles and ROS generation within 3D-cultured cells often diverge significantly from their 2D counterparts. By capturing these multifaceted aspects of TNBC biology, our 3D HA-EAbuK-IKVAV scaffolds enhance the predictive validity of in vitro screenings, offering a more reliable bridge to animal studies and, ultimately, clinical translation [18–20, 51]. This approach is clinically relevant, given TNBC’s heterogeneity, which necessitates the use of higher-fidelity platforms to characterize and target its distinct subtypes.

### Electroporation-Enhanced Resveratrol Efficacy

A key finding of this study is that transient membrane permeabilization via electric pulses potentiates the cytotoxic effects of resveratrol in TNBC cells. Despite the differences between our two electroporation protocols—EP1 employing a electric field (800 V/cm) versus EP2 using a electric field (1000 V/cm)—both regimes substantially enhanced intracellular resveratrol uptake and promoted cell death. The mechanistic underpinnings of this synergy can be attributed primarily to two factors:

1. **Increased Resveratrol Uptake**: Electroporation mechanically perturbs the lipid bilayer, thus circumventing the selective permeability of the plasma membrane and any transporters that might limit resveratrol’s intracellular accumulation [25, 27]. This phenomenon is particularly crucial for hydrophobic or partially hydrophilic molecules, like resveratrol, whose diffusion can otherwise be restricted by membrane efflux pumps or passive diffusion barriers.
2. **ROS Amplification**: Once resveratrol is internalized, its known capability to modulate oxidative metabolism can be fully engaged. By increasing reactive oxygen species (ROS)—in part through mitochondrial respiratory chain perturbations—resveratrol promotes oxidative stress conditions that undermine cancer cell survival [9, 12]. Under such stress, the mitochondrial membrane potential may collapse, triggering caspase activation and/or autophagic processes.

The 3D scaffold did not abolish the cytotoxic benefit of combining electroporation with resveratrol. EP + resveratrol produced a marked reduction in viability in both 2D and 3D cultures, indicating that electroporation effectively potentiated resveratrol activity under both culture conditions. However, ROS accumulation was lower in 3D than in 2D, suggesting that the 3D microenvironment modulates the oxidative-stress response rather than simply enhancing or suppressing treatment efficacy. This difference may reflect cell–ECM interactions, diffusion limitations, scaffold-associated gradients, and altered local electric-field distribution. Because direct measurements of intracellular resveratrol uptake, local electric-field distribution, and pore formation were not performed, these mechanisms should be interpreted cautiously. Overall, our data support the conclusion that 3D culture modifies the biological response to electroporation-enhanced resveratrol therapy, with strong cytotoxicity maintained despite a comparatively attenuated ROS signal.

### Translational Implications

Electroporation-based therapies collectively referred to as ECT are already in clinical use for cutaneous malignancies, including metastatic melanoma, and have shown promise in soft-tissue sarcomas and liver metastases [30–32].The technology’s versatility lies in its capacity to deliver a broad range of agents directly into tumor cells with minimal systemic distribution. Resveratrol’s wide-ranging molecular targets and robust safety profile make it an especially compelling candidate for these protocols, particularly in refractory cancers such as TNBC, which are often characterized by resistance to traditional chemotherapeutics [13, 52].

From a translational standpoint, our 3D scaffold model can inform critical dosing parameters such as the electric-field strength, pulse duration, and resveratrol concentration—tailored to maximize cytotoxicity while sparing healthy tissue. This is of increasing interest as emerging data suggest that combining electroporation with natural compounds or repurposed drugs may sidestep some of the severe off-target effects observed with conventional chemotherapy [25, 33, 53]. Additionally, coupling this strategy with real-time imaging of ROS dynamics and integrin signaling could illuminate the spatiotemporal progression of cell death in high-fidelity tumor models.

Beyond localized tumor control, the implications may extend toward systemic immune engagement. Electroporation-facilitated tumor cell death often elicits immunogenic cell death (ICD) markers, including calreticulin surface exposure and adjuvant-like release of damage-associated molecular patterns (DAMPs) [54]. This phenomena was observed prevalently with irreversible electroporation [55, 56], e.g. in [57] using 99 pulses at 1250 V/cm 50 µs long at 1 Hz or applying an electric field ranging between 1000 and 1500 V/cm with 200 pulses 200 µs long. Nevertheless, reversible electroporation (e.g. 8 pulses 100µs long at 1000 V/cm [36]) in combination with immunostimulant seem to be promising [58]. Resveratrol has also been implicated in modulating immunomodulatory cytokine production, raising the possibility of a dual effect whereby local therapy fosters a more robust anti-tumor immune response systemically [13, 52]. This angle is particularly relevant given the recent surge in immunotherapies for breast cancer and the need to convert TNBC’s immunologically “cold” phenotype into one more amenable to immune checkpoint inhibitors.

Future investigative directions could expand upon these findings by incorporating patient-derived TNBC organoids or patient-derived xenograft (PDX) models into the 3D electroporation framework. Additionally, mechanistic studies evaluating DNA damage pathways, autophagic flux, and cell-cycle perturbations would yield a more granular understanding of how resveratrol and electric-field modulation intersect to induce cell death. Identification of potential biomarkers that predict electroporation-resveratrol sensitivity, such as specific integrin profiles or redox regulatory proteins, could further facilitate patient stratification in clinical trial designs.

Ultimately, our data reinforce the concept that recapitulating the tumor’s 3D architecture is indispensable for unveiling the full therapeutic potential of combination strategies in TNBC. By systematically interrogating how electroporation modulates the intracellular pharmacokinetics of resveratrol and accentuates its oxidative and cytostatic effects.

## Conclusions

In this research, 3D and 2D cell culture studies of electroporation-based Resv were performed. It was demonstrated that a 3D HA-EAbuK-IKVAV scaffold provides a biologically relevant TNBC in vitro platform and enables clear quantification of electroporation-enhanced resveratrol efficacy. Across conditions, EP alone caused a moderate reduction in viability (~ 69–80% of control), whereas, resveratrol alone (100 µM) reduced viability to ~ 55–58%. Importantly, the combined EP+resveratrol regimen produced the strongest effect, reducing viability to ~ 20–35% (depending on EP protocol and culture format) and increasing oxidative stress to ~ 239–356% ROS relative to untreated controls. These results support a mechanism in which EP increases cell death and intensifies ROS-driven cytotoxicity. Overall, coupling a biomimetic HA-rich 3D culture with EP provides a practical preclinical strategy to optimize localized delivery of redox-active anticancer compounds for TNBC, with the added advantage of testing therapy under microenvironmental constraints that are absent in standard 2D assays.

Future research efforts can build on these insights in several ways. Paramount among them is the optimization of pulse parameters—voltage, pulse duration, and frequency—to refine electroporation protocols tailored for diverse TNBC phenotypes, potentially guided by computational modeling and high-throughput testing in organoids or patient-derived xenografts. In parallel, a more comprehensive exploration of molecular readouts, such as DNA damage, immune-related biomarkers, and the involvement of specific signaling networks (e.g., MAPK, PI3K–AKT, NF-κB), will help delineate the full mechanistic scope of electroporation–resveratrol synergy. Finally, validating the efficacy of this 3D-based electroporation strategy in clinically relevant, patient-derived TNBC models or ex vivo tissue explants will be a critical step toward bridging the gap between promising in vitro outcomes and real-world applications. In conclusion, the dual strategy of leveraging a biomimetic 3D scaffold and exploiting electroporation for enhanced resveratrol delivery holds substantial promise for the development of more effective and less toxic therapeutic regimens against TNBC.

## Supplementary Information

Below is the link to the electronic supplementary material.

Supplementary Material 1

Supplementary Material 2

## Data Availability

Conceptualization, P.G., R.S., E.S.; methodology, P.G., R.S., E.S., I.C.,M.C., M.D.,A.Z.; validation. P.G., A.Z.nN.M.; investigation, P.G.,A.Z., E.S. , N.M. ; data curation, P.G., E.S. , N.M. ;, writing—original draft preparation, P.G., E.S., N.M.,M.D. ; writing—review and editing, R.S., E.S.,L.D. ; supervision I.C., R.S. All authors have read and agreed to the published version of the manuscript. All data supporting the findings of this study are available within the paper.
